# Demographic, Social, and Behavioral Determinants of Lung Cancer Perceived Risk and Worries in a National Sample of American Adults; Does Lung Cancer Risk Matter?

**DOI:** 10.3390/medicina54060097

**Published:** 2018-12-03

**Authors:** Hamid Chalian, Pegah Khoshpouri, Shervin Assari

**Affiliations:** 1 Department of Radiology, Duke University Medical Center, Durham, NC 27705, USA; 2 Russell H. Morgan Department of Radiology and Radiological Sciences, Johns Hopkins University School of Medicine, Baltimore, MD 21205 USA; pkhoshp1@jhmi.edu; 3 Department of Psychiatry, University of Michigan, Ann Arbor, MI 48104, USA; assari@umich.edu; 4 Department of Psychology, University of California, Los Angeles (UCLA), Los Angeles, CA 90095, USA

**Keywords:** perceived risk, worries, lung cancer screening, Health Information National Trends Survey (HINTS)

## Abstract

*Background*: Perceived risk and worries of developing cancer are important constructs for cancer prevention. Many studies have investigated the relationship between health behaviors and subjective risk perception. However, factors correlated with lung cancer risk perception and worries in individuals more susceptible to lung cancer have rarely been investigated. *Objective*: To determine demographic, social, and behavioral determinants of cancer perceived risk and worries and to explore heterogeneities in these associations by the level of lung cancer risk in a nationally representative sample of American adults. *Methods*: For this cross-sectional study, data came from the Health Information National Trends Survey (HINTS) 2017, which included a 2277 representative sample of American adults. Smoking status, cancer perceived risk, cancer worries, age, gender, race, education, income, and insurance status were measured. We ran structural equation models (SEMs) for data analysis. *Results*: “Ever smoker” status was associated with higher cancer perceived risk (b = 0.25; 95% CI = 0.05–0.44, *p* = 0.013) and worries (b = 0.34, 95% CI = 0.18–0.50, *p* < 0.001), suggesting that “ever smokers” experience higher levels of cancer perceived risk and worries regarding cancer, compared to “never smokers”. Other factors that correlate with cancer perceived risk and worries were race, age, income, and insurance status. Blacks demonstrated less cancer perceived risk and worry (b = −0.98, 95% CI = −1.37–0.60, *p* < 0.001) in both low and high risk lung cancer groups. However, the effects of social determinants (income and insurance status) and age were observed in low but not high risk group. *Conclusions*: Determinants of cancer perceived risk and worries vary in individuals depending on the level of lung cancer risk. These differences should be considered in clinical practice and policy makings with the goal of improving participation rates in lung cancer screening programs.

## 1. Introduction

With an estimated 234,030 new cases and 154,050 cancer-related deaths in 2018, lung cancer is the leading cause of cancer death for both genders in the United States [1]. A low overall five-year relative survival rates of 24% in women and 17% in men are due to the fact that most lung cancer cases are diagnosed in advanced stages of disease [2]. This highlights the need for increased lung cancer screening programs nationwide.

The National Lung Cancer Screening Trial (NLST), a randomized clinical trial including more than 50,000 participants, showed a 20% decrease in lung cancer death and 6.7% decrease in all-cause mortality using annual low-dose computed tomography screening [3]. This important data and several other smaller reports shed light on the importance of lung cancer screening [4,5]. Based on these reports, several related organizations, including the U.S. Preventive Services Task Force (USPSTF), American Cancer Society, American College of Chest Physicians, and American College of Radiology, issued recommendations and guidelines for annual lung cancer screening using low-dose computed tomography imaging in high risk patients [6,7,8]. In February 2015, the Center for Medicare and Medicaid Services (CMS) approved lung cancer screening using low-dose computed for high risk beneficiaries between the age of 55 and 77 years who have a 30-pack-year smoking history and currently smoke or have quit within the past 15 years [9].

Unfortunately, participation in lung cancer screening programs has always been low even after the CMS approved coverage for low-dose computed tomography screening. A recent American Cancer Society study found that only 3.9% of the current and former smokers eligible for lung cancer screening in 2015 received lung cancer screening [10]. Understanding the causes of this behavior is crucial for better planning to improve lung cancer screening participation. Risk perception is a key predictor of health behaviors [11]. The social context in which behavior occurs is evolving. Therefore, demographic and social determinants (such as race, age, gender, and income) that were useful in the past, as determined in the context of more stablished cancer screening programs than lung cancer screening, may be of limited use today. Public health programs should be consistently refined based on the new epidemiological information and social science research [12].

Although there are reports on the perception of lung cancer in the general population and smokers [13,14], characterizing cancer risk perception and worries is relatively underdeveloped for specific cancer types including lung cancer [15]. Furthermore, no report has been published on the cancer risk perception and worries of individuals at risk for lung cancer and their correlates in individuals who are candidates for lung cancer screening as defined by CMS guidelines. Knowing the correlates of cancer risk perception and worries might be helpful in better understanding the causes of low lung cancer participation in high risk groups. To investigate characteristics that might improve participation in lung cancer screening programs, we aimed to determine demographic, social, and behavioral factors correlated with the lung cancer perceived risk and worries in the American adult population. We also aimed to assess the effect of lung cancer risk defined by the CMS guideline [9] on cancer perceived risk and worries correlates.

## 2. Methods

### 2.1. Design and Setting

This cross-sectional study used data from the 2017 Health Information National Trends Survey (HINTS-5). Periodically administered by the National Cancer Institute (NCI) since 2003, HINTS is a nationally representative survey. The purpose of HINTS is to provide a national picture of cancer information among American adults [16]. Data of the HINTS-5-Cycle 1 were collected from January 2017 through May 2017.

### 2.2. Ethical Considerations

The HINTS-5 study protocol was approved by the Westat’s Institutional Review Board (IRB) (Westat’s Federalwide Assurance (FWA) number is FWA00005551 and Westat’s IRB number is 00000695. The project used to have an OMB number (0920-0589). The NIH Office of Human Subjects did exempt the HINTS study from IRB review. All participants provided informed consent.

### 2.3. Sampling

The HINTS target population is non-institutionalized American adults (age ≥18) who reside in the United States. HINTS-5-Cycle 1 used a two-stage sampling design. First stage of the sampling was a stratified sample of addresses that were derived from all residential addresses received from the Marketing Systems Group (MSG). All non-vacant residential addresses were considered eligible for sampling. In the second stage of the sampling, one adult was selected from each sampled household. The sampling frame was grouped into two strata: Stratum #1, areas with a high concentration of minorities, and Stratum #2, areas with a low concentration of minorities. Equal-probability sampling was used to draw addresses from each sampling stratum [16].

### 2.4. Surveys

The surveys were sent to the participants by mail. Monetary incentive was included in the mails to encourage participation. Two toll-free telephone numbers were provided to respondents: one was used for English calls and one was used for Spanish calls. The overall response rate was 32.4 percent [16].

### 2.5. Study Variables

The study variables included race, ethnicity, age, gender, education, income, smoking status, health insurance status, and cancer perceived risk and cancer worries.

### 2.6. Independent Variables

*Smoking Status*. Smoking status was measured using the following item: “Have you smoked at least 100 cigarettes in your entire life?”. Response options for this question were yes and no. “Ever smoker” status was defined as a positive response to this question.

*Demographic Factors.* Race, ethnicity, age, and gender were measured. Race was a dichotomous variable (0 Whites, 1 Blacks). Ethnicity was Hispanic versus Non-Hispanic. Age was a continuous measure ranging from 18 to 101. Gender was a dichotomous variable (0 female, 1 male).

*Socioeconomic Status (SES).* SES indicators in this study included education and income. Education attainment was measured as an ordinal variable with the following five categories: (1) Less than high school, (2) high school graduate, (3) some college, (4) bachelor’s degree, and (5) post-baccalaureate degree. In this study, education attainment was operationalized as a continuous measure, ranging from 1 to 5, with a higher score reflecting higher educational attainment. Household income was measured using a five level ordinal variable: (1) Less than $20,000, (2) $20,000–34,999, (3) $35,000–49,999, (4) $50,000–74,999, and (5) $75,000 or more. Household income was also treated as a continuous measure, ranging from 1 to 5, with higher scores indicating higher household income.

*Health Insurance.* Health insurance status was evaluated using the following types of insurance: (1) Insurance purchased directly from an insurance company, (2) Medicare, for people 65 and older, or people with certain disabilities, (3) Medicaid, Medical Assistance, or any kind of government-assistance plan, (4) TRICARE or other military health care, (5) veterans affairs (VA, including those who have ever used or enrolled for VA health care) (6) Indian Health Service and (7) Any other type of health insurance or health coverage plan. Insurance status was treated as a dichotomous variable (0 without insurance, 1 with insurance).

### 2.7. Dependent Variables

Two single items were used to measure cancer perceived risk and cancer worries. Cancer perceived risk was measured using the following item: “How likely are you to get cancer in your lifetime?”. Responses included (1) Very unlikely, (2) Unlikely, (3) Neither unlikely nor likely, (4) Likely, and (5) Very likely. Cancer worries were measured using this item: “How worried are you about getting cancer?”. Responses included (1) Not at all, (2) Slightly, (3) Somewhat, (4) Moderately, and (5) Extremely. Both variables were operationalized as continuous measures, with a potential range score from 1 to 5. For both items, a higher score indicated a worse condition (more cancer perceived risk or more cancer worries) [17,18].

### 2.8. Effect Modifier

*Lung Cancer Risk.* Ages between 55 and 77 years and ever smoking status were used to group participants into the following two risk groups: High risk group, ages between 55 and 77 years and positive history of ever smoking. Low risk group, any other individual. This categorization was based on recommendations of the CMS for identification of high risk beneficiaries for the lung cancer screening program [9]. Since pack-year smoking history was not documented in the HINTS dataset, we could not adjust exactly based on pack-year smoking.

### 2.9. Statistical Analysis

We used Stata 15.0 (Stata Corp., College Station, TX, USA) for univariate, bivariate, and multivariable analyses. For univariate analysis, we reported mean, frequencies, and their standard errors. For bivariate associations, Pearson’s correlations tests, independent sample *t*-tests, and paired *t*-tests were used. To test demographic, social, and behavioral determinants of perceived risk of cancer and cancer worries, we ran multi-group structural equation modeling (SEM) [19] where groups were defined based on the level of lung cancer risk. Perceived risk of cancer and cancer worries were dependent variables and race, gender, ethnicity, age, education, income, insurance, and smoking status were independent variables. To test the effects of smoking status on cancer perceived risk and worries, we ran models in the pooled sample, as well as based on the level of risk. We reported path coefficients, SE, 95% CI, z-value, and p-value. *P* < 0.05 was considered significant.

We used maximum likelihood estimates in the presence of missing data [20,21]. Conventional model fit statistics such as the comparative fit index (CFI) and the root mean square error of approximation (RMSEA) were used to evaluate the goodness of fit. A chi-square to degrees of freedom ratio of less than 4, a CFI above 0.95, and a RMSEA value of 0.06 or less were considered as indicators of good fit of the data [22,23].

## 3. Results

### 3.1. Descriptive Statistics

The mean age of the participants was 49 years (SE = 0.34), and 52% of the participants were females. Thirteen percent of the participants were Black. About 92% of the participants had insurance. Table 1 provides a summary of the descriptive statistics for the pooled sample and subgroups based on lung cancer risk.

### 3.2. Determinants in the Pooled Sample

**Figure 1:**
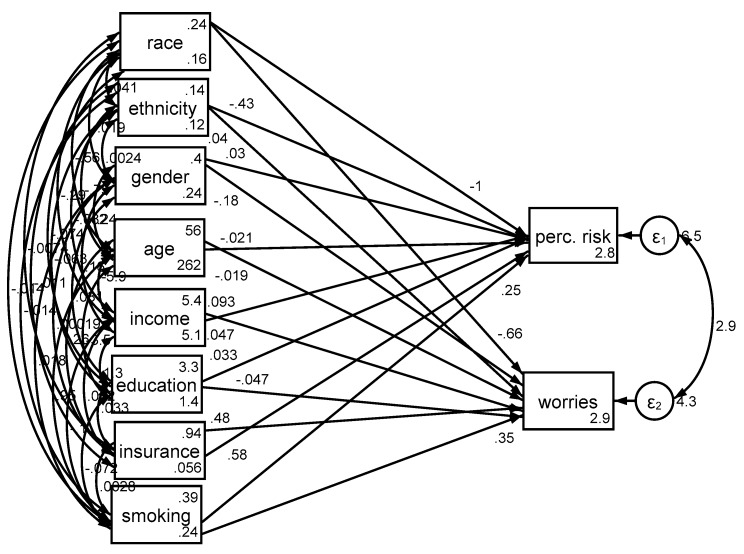
Structural equation models in the pooled sample (n = 2277).

*Cancer perceived risk.* In the pooled sample, race was associated with cancer perceived risk (b = −0.98, 95% CI = −1.37–0.60, *p* < 0.001), with Blacks reporting lower perceived risk of cancer compared to Whites. While older age was associated with lower cancer perceived risk (b = −0.02; 95% CI = −0.03–0.01, *p* < 0.001), gender was not associated with cancer perceived risk (*p* > 0.05). While high income was associated with higher cancer perceived risk (b = 0.09, 95% CI = 0.03–0.15, *p* = 0.002), education was not associated with the same outcome (*p* > 0.05). Having insurance was also associated with higher cancer perceived risk (b = 0.58, 95% CI = 0.19–0.96, *p* = 0.003). There was a positive and significant path from ever smoking status to cancer perceived risk (b = 0.25; 95% CI = 0.05–0.44, *p* = 0.013), suggesting that individuals who were ever smokers experienced more cancer perceived risk compared to their never smoker individuals (Table 2, Figure 1).

*Cancer worries.* In the pooled sample, race was associated with cancer worries (b = −0.48, 95% CI −0.78–0.18, *p* = 0.002), with Blacks reporting lower levels of worries about cancer compared to Whites. High age (b = −0.02, 95% CI = −0.03–0.01, *p* < 0.001) was associated with less cancer worries, but high income (b = 0.06, 95% CI = 0.01–0.11, *p* = 0.014) and having insurance (b = 0.44, 95% CI = 0.13–0.75, *p* = 0.005) were associated with more cancer worries (Table 2, Figure 1).

**Figure 2:**
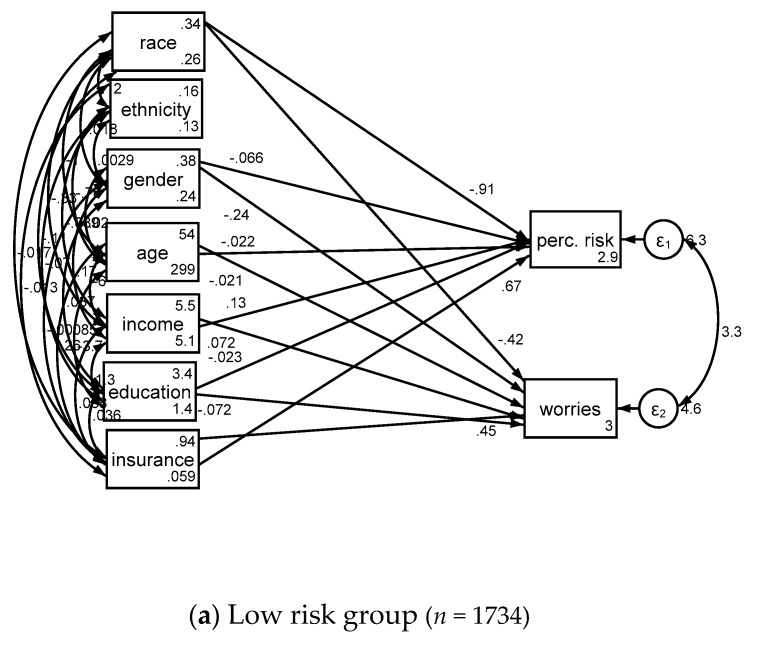

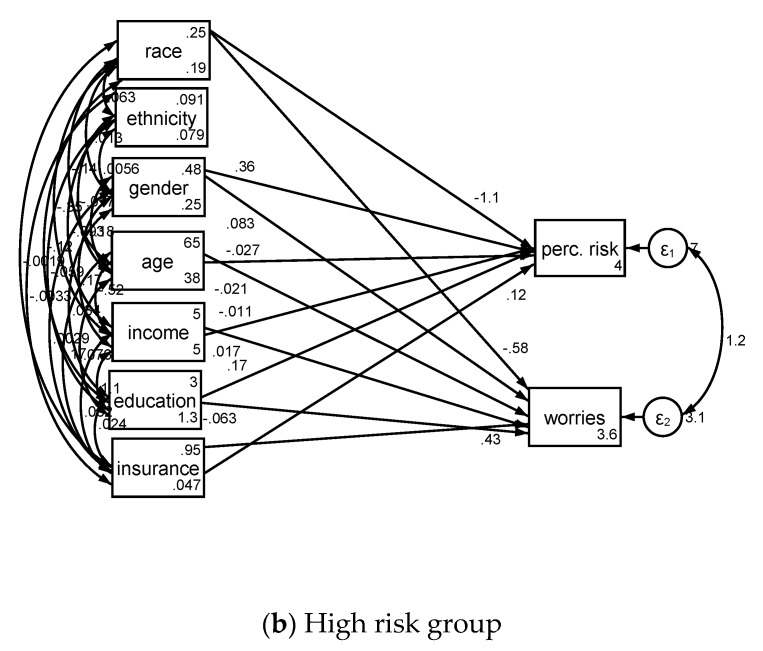
Structural equation models in low risk (**a**) and high risk (**b**) groups.

Associates of cancer perceived risk and worries based on lung cancer risk is shown in Table 3 and Figure 2.

## 4. Discussion

We found that being Black and of old age were associated with lower cancer perceived risk and worries irrespective of lung cancer risk. However, the effects of income and having insurance on cancer perceived risk and worries were conditional on cancer risk as they were only seen in individuals with low risk of lung cancer. Gender, ethnicity, and education were not correlates of cancer perceived risk or cancer worries irrespective of lung cancer risk level.

Smokers experienced higher levels of cancer perceived risk and worries in our analysis. These results are in line with the results of HINTS 2005 [14] regarding a positive association between smoking and cancer perceived risk. We further analyzed a large cohort of a random US population to identify the determinants of cancer perceived risk and worries in subgroups with high and low lung cancer risk.

Racial minority status, particularly being Black, is shown to be associated to lower cancer perceived risk and cancer worries [24], which may be due to low cancer literacy [25]. This is paradoxical and undesired because Blacks are at an increased risk of many types of cancer [26]. The finding that Blacks have lower cancer perceived risk and cancer worries was persistent in both high and low lung cancer risk groups. There is a need to address racial disparities in cancer perceived risk, as it may be one mechanism explaining racial gap in lung cancer survival rate [27]. It has been shown that despite coverage provided through the Affordable Care Act, Black patients are less likely to qualify for lung cancer screening [28]. In a cross-sectional study performed in the state of Indiana on 438 long-term smokers, racial and geographic disparity has been show in lung cancer screening participation [29]. Being White has also been shown to be an independent associate of high risk perception in a data collected from 630 national lung screening trial participants [30]. Racial disparity in perceived risk and worries of lung cancer among those with high risk for contracting this disease have implications in policy making, with the goal of increasing participation of high risk black individuals in lung cancer screening programs.

We found a negative correlation between age and cancer perceived risk and worries in the whole cohort and low lung cancer risk group. In a cross-sectional study on lung cancer-eligible patients, age was not a significant factor affecting lung cancer screening programs [29]. In a qualitative study assessing attitudes to participation in lung cancer screening, “being too old to benefit from lung cancer screening” was among the causes of declining lung cancer screening participations [31]. Older age has been among the determinants of declining participation in lung cancer screening in the UK [32]. Although age increases the risk of cancer, and many health problems including cancers are age related [33,34], aging may be associated with a mental discounting of perceived risk and worries about cancer. A previous meta-analysis has shown in most studies, age is negatively correlated with cancer perceived risk; however, the effect size is small [35]. This is in line with our findings, which show less cancer worry and perceived risk in older individuals. Our further analysis demonstrates that the effect of age on cancer perceived risk and worry disappears in the lung cancer high risk group and persists in the lung cancer low risk group. This is an interesting finding which has not been reported before. Note that what we see in this study as the effect of age may be in fact a cohort effect [36]. It is very difficult to separate age and cohort effect which requires longitudinal data with multiple observations [37].

In our analysis, women showed more cancer worries only in the low risk group. Men and women in the high risk group had similar levels of cancer worry and cancer perceived risk. In a study on long-term smokers eligible for lung cancer screening program, gender was not significantly different in screening versus non-screening groups [29]. Gender is shown to be a salient determinant of perceived risk across domains [38,39] including cancer risk [34]. Not only in cancer related worries but all types of worries are more common in women than men [40]. The same pattern of worries are shown in a wide range of health domains [41] and holds for sub-clinical and clinical levels of anxiety, fear, and worries [42,43]. This may be in part due to gendered socialization and upbringing [44]. The fact that there is no gender disparity in cancer perceived risk and worries in high risk group might suggest that health system appropriately increases lung cancer awareness in this group.

High income was found to be associated with higher cancer perceived risk and worries in the whole cohort. However, further analysis demonstrated that income may not determine cancer perceived risk and worries in the high risk for lung cancer group, but is still a significant determinant for low risk for lung cancer group. It has been shown that low income group are less likely to participate in lung cancer screening programs [29]. It is paradoxical that SES (high income in this analysis) is associated with higher cancer perceived risk and worries [25] while in fact high SES is protective against cancer risk behaviors, such as smoking, being overweight, and low physical activity [45]. Concerns of high SES individuals about cancer might be related to exposure to health literacy, shaped by messages and media, and communication of health care providers, or may be simply due to a tendency to have more concerns about one’s own health [46].

To avoid unnecessary and over adjustment [47,48], we decide not to control for quality of life and health behaviors. Quality of life is a broad measure which is correlated with most of the variables and constructs in this study as well as many others. For example, quality of life correlates with age, gender, socioeconomic status, health, cancer risk, and perceived risk of cancer [49]. Health behaviors are also correlated with actual and perceived risk of cancer [50]. While health behaviors and quality of life possibly correlate with our independent and dependent variables, controlling for them would have biased our results toward the null, as they could partially mediate our associations of interest. To reduce the risk of bias, researchers should be cautious about omitted confounders as well as not to control for potential mediators that are involved in underlying mechanisms [47,48]. Causal directed acyclic graphs (DAGs) can be a useful guide for selection of confounders in future research [51,52,53,54].

### 4.1 Limitations

Our study had a few limitations. We did not assess lung cancer screening participation rate or predictors of lung cancer screening participation. We did not include a comprehensive measure of cancer perceived risk. This analysis did not differentiate between absolute and comparative perceived risk [55,56]. Although we could not generate the exact lung cancer high risk population based on the CMS guideline due to a lack of measurement of pack-year smoking in HINTS, we included individuals between 55 and 77 years old with a history of smoking as per CMS guidelines. Considering the fact that the mean age was 49 years in our cohort and most smokers initiate smoking prior to age 26, it is probable that the majority of the smokers in our cohort are long-term smokers [57]. The cross-sectional nature of our data and the use of self-report measures are also among the limitations of our analysis. Despite these limitations, large sample size, national representative sample, and conceptualization of risk as a moderator were among the strengths of this study.

### 4.2. Implications

Although we did not directly assess the effect of socioeconomic factors on lung cancer screening participation, the results of our analysis might have significant implications for practice and policy-making to improve participation of individuals at high risk for lung cancer into a lung cancer screening program. First, fewer determinants are available in high lung cancer risk group. This makes the promotion of cancer screening in high risk group more difficult and probably costlier. Second, older individuals, minorities, and low SES people, although at an increased risk of lung cancer, paradoxically reported low cancer perceived risk. These people may be less willing to undergo screening for something they do not find as a risk or threat. Efforts for education are needed and may require messages by clinicians as well as media campaigns, particularly for high risk groups. Third, as smokers already perceive the risk, they may have higher readiness to participate into lung cancer screening programs.

## 5. Conclusions

We found that determinants of cancer perceived risk and worries vary in adults with low versus high lung cancer risk. Income and insurance are correlated with higher cancer perceived risk and worries in low lung cancer risk group but not in the high lung cancer risk group. Being Black was associated with lower cancer perceived risk and worries in both low and high lung cancer risk groups. While cancer perceived risk and worries reduce quality of life and cause distress [50], these constructs can be leveraged to promote lung cancer screening participation. More research is needed on the most efficient strategies to improve lung cancer screening participation in the high risk group. 

## Figures and Tables

**Table 1:** Descriptive statistics in the pooled sample and by lung cancer risk level.

	All (*n* = 2277)		Low Risk (*n* = 1734)		High Risk (*n* = 543)	
	% (SE)	95% CI	%(SE)	95% CI	%(SE)	95% CI
Race						
Whites	86.66 (0.01)	85.48–87.85	85.90(0.01)	84.53–87.28	90.83(0.01)	87.84–93.82
Blacks	13.34 (0.01)	12.15–14.52	14.10 (0.01)	12.72–15.47	9.17 (0.01)	6.18–12.16
Gender						
Male	47.89 (0.01)	46.57–49.21	47.03 (0.01)	45.50–48.57	52.58 (0.02)	48.61–56.55
Female	52.11 (0.01)	50.79–53.43	52.97 (0.01)	51.43–54.50	47.42 (0.02)	43.45–51.39
Health Insurance						
No	7.87 (0.01)	6.40–9.35	8.36 (0.01)	6.62–10.10	5.23 (0.01)	2.27–8.18
Yes	92.13 (0.01)	90.65–93.60	91.64 (0.01)	89.90–93.38	94.77 (0.01)	91.82–97.73
	**Mean (SE)**	**95% CI**	**Mean (SE)**	**95% CI**	**Mean (SE)**	**95% CI**
Age	48.88 (0.34)	48.19–49.56	46.43 (0.38)	45.65–47.20	64.15 (0.28)	63.58–64.72
Income	5.60 (0.05)	5.49–5.70	5.67 (0.06)	5.55–5.78	5.16 (0.14)	4.88–5.44
Education	3.12 (0.02)	3.08–3.16	3.18 (0.02)	3.13–3.22	2.80 (0.06)	2.68–2.91
Cancer Perceived Risk	2.93 (0.02)	2.83–3.03	2.96 (0.05)	2.86–3.06	2.75 (0.17)	2.41–3.10
Cancer Worries	2.54 (0.04)	2.45–2.62	2.55 (0.05)	2.45–2.65	2.48 (0.07)	2.34–2.63

Notes. Source: Health Information National Trends Survey (HINTS-5), 2017. CI, Confidence Interval; SE, Standard Error.

**Table 2:** Results of path analysis in the pooled sample (*n* = 2277).

		B (SE)	95% CI	*z*	*p*
Ever smoker	Cancer perceived risk	0.25 (0.10)	0.05–0.44	2.49	0.013
Race (Blacks)	Cancer perceived risk	−0.98 (0.20)	−1.37–0.60	−5.03	<0.001
Gender (Male)	Cancer perceived risk	0.03 (0.10)	−0.17–0.23	0.31	0.757
Age	Cancer perceived risk	−0.02 (0.00)	−0.03–0.01	−6.18	<0.001
Income	Cancer perceived risk	0.09 (0.03)	0.03–0.15	3.12	0.002
Education	Cancer perceived risk	0.03 (0.05)	−0.07–0.13	0.61	0.544
Health Insurance	Cancer perceived risk	0.58 (0.20)	0.19–0.96	2.94	0.003
Intercept	Cancer perceived risk	2.83 (0.35)	2.15–3.51	8.17	<0.001
Ever smoker	Cancer worries	0.34 (0.08)	0.18–0.50	4.15	<0.001
Race (Blacks)	Cancer worries	−0.48 (0.15)	− 0.78–0.18	−3.11	0.002
Gender (Male)	Cancer worries	−0.16 (0.08)	−0.33–0.00	−1.95	0.051
Age	Cancer worries	−0.02 (0.00)	−0.03–0.01	−6.86	<0.001
Income	Cancer worries	0.06 (0.03)	0.01–0.11	2.24	0.025
Education	Cancer worries	−0.06 (0.04)	−0.14–0.03	−1.37	0.170
Health Insurance	Cancer worries	0.44 (0.16)	0.13–0.75	2.81	0.005
Intercept	Cancer worries	2.91 (0.29)	2.34–3.47	10.04	<0.001

Notes. Source: Health Information National Trends Survey (HINTS-5), 2017. CI, Confidence Interval; SE, Standard Error; Z, Z score

**Table 3:** Results of path analysis based on the risk level.

		Low Risk Group (*n* = 1734)				High Risk Group (*n* = 543)			
		B (SE)	95% CI	*z*	*p*	B (SE)	95% CI	*z*	*p*
Race (Blacks)	Cancer perceived risk	−0.91 (0.24)	−1.38–0.44	−3.79	<0.001	−1.13 (0.34)	−1.80–0.46	−3.29	0.001
Gender (Male)	Cancer perceived risk	−0.07 (0.12)	−0.29–0.16	−0.57	0.568	0.36 (0.21)	−0.05–0.77	1.70	0.089
Age	Cancer perceived risk	−0.02 (0.00)	−0.03–0.01	−6.02	<0.001	−0.03 (0.02)	−0.06–0.01	−1.65	0.099
Income	Cancer perceived risk	0.13 (0.04)	0.06–0.20	3.66	<0.001	−0.01 (0.06)	−0.13–0.11	−0.18	0.855
Education	Cancer perceived risk	−0.02 (0.06)	−0.14–0.09	−0.39	0.694	0.17 (0.10)	−0.03–0.37	1.64	0.101
Health Insurance	Cancer perceived risk	0.67 (0.21)	0.25–1.10	3.15	0.002	0.12 (0.47)	−0.81–1.05	0.25	0.800
Intercept	Cancer perceived risk	2.85 (0.38)	2.11–3.60	7.48	<0.001	3.99 (1.18)	1.68–6.30	3.39	0.001
Race (Blacks)	Cancer worries	−0.42 (0.18)	−0.77–0.07	−2.35	0.019	−0.58 (0.22)	−1.01–0.16	−2.67	0.008
Gender (Male)	Cancer worries	−0.24 (0.10)	−0.44–0.05	−2.41	0.016	0.08 (0.14)	−0.19–0.36	0.59	0.553
Age	Cancer worries	−0.02 (0.00)	−0.03–0.02	−6.65	<0.001	−0.02 (0.01)	−0.04–0.00	−1.89	0.058
Income	Cancer worries	0.07 (0.03)	0.01–0.13	2.33	0.020	0.02 (0.04)	−0.06–0.09	0.43	0.668
Education	Cancer worries	−0.07 (0.05)	−0.17–0.03	−1.40	0.161	−0.06 (0.07)	−0.20–0.07	−0.90	0.367
Health Insurance	Cancer worries	0.45 (0.18)	0.10–0.81	2.49	0.013	0.43 (0.31)	−0.18–1.05	1.38	0.168
Intercept	Cancer worries	3.03 (0.33)	2.37–3.69	9.04	<0.001	3.58 (0.78)	2.05–5.11	4.58	<0.001

Notes. Source: Health Information National Trends Survey (HINTS-5), 2017. CI, Confidence Interval; SE, Standard Error; Z, Z score
